# Orbital angular momentum light interacted with double quantum dot-metal nanoparticle hybrid structure under spontaneous coherence

**DOI:** 10.1038/s41598-025-86417-w

**Published:** 2025-01-27

**Authors:** Mohanad Ahmed Abdulmahdi, Amin Habbeb Al-Khursan

**Affiliations:** 1 Department of Physics, College of Science, University of Thi-Qar, Nasiriya, Iraq; 2 Nasiriya Nanotechnology Research Laboratory (NNRL), College of Science, University of Thi- Qar, Nasiriya, Iraq

**Keywords:** Double quantum dot, Metal nanoparticle, Orbital angular momentum, Materials science, Nanoscience and technology, Optics and photonics

## Abstract

This work studies the generation of the orbital angular momentum (OAM) beam in the double quantum dot-metal nanoparticle (DQD-MNP) system under the application of the OAM beam. First, an analytical model is derived to attain the relations of probe and generated fields as a distance function in the DQD-MNP system under OAM applied field and spontaneously generated coherence (SGC) components. The calculation here is of material property; it differs from others by calculating energy states of the DQDs and the computation of the transition momenta between quantum dot (QD)-QD and QD-wetting layer (WL) transitions. The orthogonalized plane wave (OPW) calculates QD-WL transitions and their momenta. The momentum calculation is essential to specify the Rabi frequency of the input field. Such characteristics are not used in earlier models. The results show that SGC is vital in increasing the generated field. The signal field generated in the DQD-MNP system doubles that from the DQD system alone. So, the DQD-MNP system is preferred to the DQD system. The generated field in the DQD-MNP for the strong coupling DQD-MNP system is higher than that for the weak coupling. Increasing the distance separating the DQD-MNP reduces the generated field. Higher OAM number reduce the generated field at a long distance in the device. The model is then extended to study the effect of incoherent pumping ($\:{R}_{inc}$) and the relations are modified to cover this part. The results show that $\:{R}_{inc}$ reduces the generated field. While the results that compare the weak and strong coupling appear for the first, others compare well to the literature.

## Introduction

The work with beams carrying orbital angular momentum (OAM) offers a new opportunity for optical applications such as optical information encoding, optical manipulation, sensing, imaging, and four-wave mixing processes^1^. The wavefront of the OAM beams is helical (twisted), such as corkscrew, spiraling along the beam direction^2^. The optical vortex beam has the intensity of a ring shape. This makes the center of the beam dark, with zero intensity^3^. Vortices of OAM states have a helical phase $\:{e}^{i\varnothing\:m}$ with $\:m$ a winding number where it is twisted by $\:2\pi\:m$ phase around the vortex. The Laguerre-Gaussian (LG) modes can be used^4^. The OAM beams destroy the electromagnetically induced transparency (EIT) at the absorption bath by the losses at the core of the vortex. Such behavior results from the zero-intensity vortex beam at the core, and a four-level atomic configuration is introduced to avoid these losses^5^. Different phenomena can occur when the optical vortex beam interacts with the atomic systems: torque induced by light, atom vortex beams, photon beam entanglement of OAM states, FWM OAM-based, spatially dependent optical transparency, and the slow-light vortex^6^.

Creating OAM light is possible in different ways, such as cylindrical lens mode converters, spiral phase plates, and spatial light modulators. The interaction of OAM light with different atomic systems is studied^7^. It is studied in Rydberg atoms, nuclear ensembles of various types, such as $\:{\Lambda\:}-$ and $\:V$-type systems^3^. Dual polarization OAM light is generated with metasurfaces^8^. Transmission of data by on-off switching of the OAM light is possible by controlling the temperature of the atomic cell^9^. Teleporting multi-optical modes using OAM light increases information transmission capacity^10^.

Quantum dot (QD) nanostructures are nanocrystals that can be used in optical and quantum applications. These artificial atoms have superior characteristics to other atomic structures. These zero-dimensional nanostructures have discrete energy states; they have a high gain and can be attached to other structures in the required application^11^. Double QD (DQD) structures are preferred over QDs because of their manipulation possibility, which gives higher performance. Surrounding QDs with metal nanoparticles (MNPs) modifies their characteristics, such as shifts in their resonance frequency and high absorbed energy yield^12^. OAM light in QD molecule is studied in^13^, where they show an intensity pattern associated with the radial index of the generated beam that differs from pure LG beams. No work studies the optical properties of the DQD-MNP system under the application of the OAM beam.

It is possible to generate coherence from two spontaneous emission components. It is built by the decay either into two lower states from the above state $\:({\Lambda\:}-$type system) or vice versa, i.e., for two-fold upper states decaying into the ground one $\:(\text{V}-$type system)^14^. Such created coherence is called spontaneously generated coherence (SGC). It occurs under non-orthogonal dipole moments of the two SGC components^15,16^.

This work studies the generation of the OAM beam in the DQD-MNP under the application of the OAM beam. First, a model is derived for the DQD-MNP system under the OAM applied field and SGC components where the relations for probe and generated fields are attained. Then, these relations are used to study the system properties. Our solution differs from others by the calculated energy states of the DQDs and the computation of the transition momenta between QD-QD and QD-wetting layer (WL) transitions. The orthogonalized plane wave (OPW) is used to calculate QD-WL transitions. It is essential to specify the Rabi frequency of the input field. Note that this work considers both the conduction band (CB) and the valence band (VB) QD and WL states; this is another property of this work. This was not considered earlier in the OAM QD works^13^, and it is essential for the work to be more realistic. So, the study of the DQD-MNP structure considering the calculation of QD-QD and WL-QD momenta under OPW, which is essential to specify Rabi frequency, characterizes this work from the literature. The way of deriving the relations differs from the literature on OAM in atomic systems. Such characteristics are not used in earlier models. It is vital to make the formulation give a picture of the practice and not use an average value or a value of one structure in another where each structure has its properties that differ from others. This makes the formulation here of material property. SGC is essential in increasing the generated field. The signal field generation in the DQD-MNP system is higher by three orders than in the DQD system alone. So, the DQD-MNP system is preferred to the DQD system. The generated field in the DQD-MNP for the strong coupling case is higher than that for the weak coupling. Increasing the distance separating the DQD-MNP system reduces the generated field. The effect of incoherent pumping is then included, where the density matrix equations system is modified to include such an impact. The results show that the generated field is increased under small incoherent injection. The results of this work make a good comparison to the literature.

## DQD-MNP structure

**Fig. 1:**
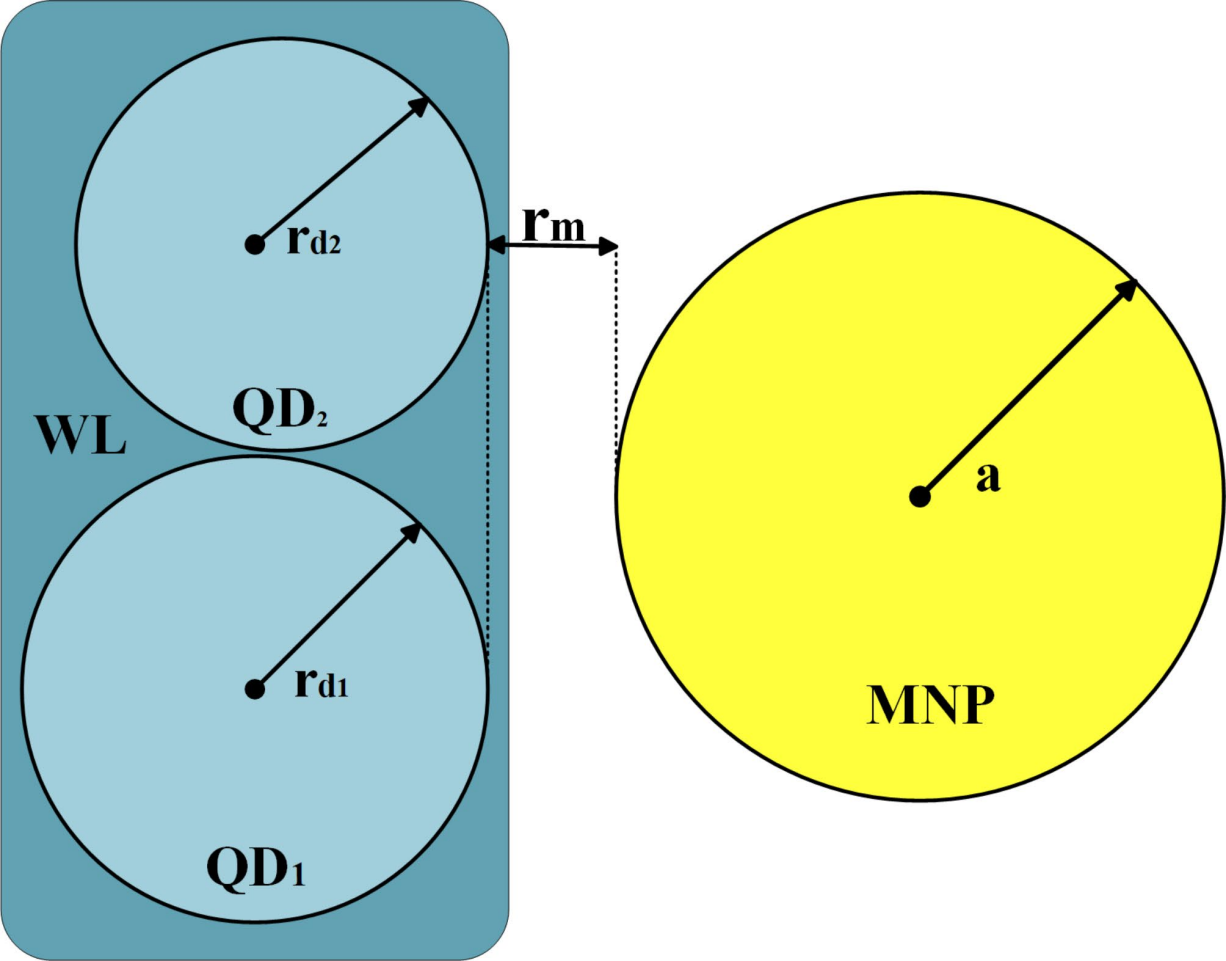
The hybrid WL-DQD-MNP structure. Note that $\:{r}_{d1}$ and $\:{r}_{d2}$ are DQD radii, $\:{r}_{m}$ is the DQD-MNP separating distance, and $\:\text{a}$ is the MNP radius.

The studied structure is a hybrid DQD-MNP structure. DQDs structure comprises two QDs composed of InAs disk-shaped QDs. These two QDs have $\:{r}_{d1}\left(=13\:nm\right),\:{h}_{d1}(=2\:\text{n}\text{m})$ as a radius and height of the first QD, while those of the second QD are $\:{r}_{d2}(=11\:nm)$,$\:\:{h}_{d2}(=3\:nm)$. The MNP is of a spherical shape of $\:{r}_{m}(=16\:nm)$ radius. The MNP-DQD distance is denoted by $\:{R}_{dm}(=6\:\text{n}\text{m})$; see Fig. 1. The strong coupling case between the MNP-DQD hybrid system is applied, i.e., $\:R<{r}_{m}$^17^. The WL is an $\:{In}_{0.5}{Ga}_{0.5}As$ quantum well of $\:9\:nm$ thickness.

## Modeling

### DQD-MNP Hamiltonian

This work considers the DQD-MNP system under the applied probe field $\:{\text{E}}_{02}\left(\text{t}\right)=\frac{{E}_{02}^{0}}{2}{e}^{-i{\omega\:}_{02}t}+\text{c}.\text{c}.$ where $\:{\omega\:}_{02}$ is the frequency of transition between $\:\left| 0 \right\rangle \leftrightarrow \:\left| 2 \right\rangle$ DQD states, $\:{E}_{02}^{0}$ is the intensity of the applied probe, and $\:\text{c}.\text{c}.$ refers to the complex conjugate. The total Hamiltonian of the system is given by,

1 $$H = H_{0} + H_{{\text{int} }} + H_{{relax}}$$

With $\:{\text{H}}_{0}$ is the unperturbed Hamiltonian, which is given by the energy of DQD states $H_{0} = \mathop \sum \limits_{{i = 0}}^{5} \hbar \omega _{i}$. The relaxation Hamiltonian $\:{\text{H}}_{relax}$ defines the relaxations of the system. The interaction Hamiltonian is given by,

2 $$\begin{aligned} \:H_{{\text{int} }} & = \left[ {\begin{array}{*{20}c} 0 & {T_{{10}} } & {\Omega_{{20}}^{{\left( 1 \right)}} } \\ {T_{{10}} } & {0} & {\beta_{{21}} } \\ {\begin{array}{*{20}c} {\Omega_{{20}}^{{\left( 1 \right)}} } \\ {\beta_{{30}} } \\ {\begin{array}{*{20}c} {\beta_{{40}} } \\ {0} \\ \end{array} } \\ \end{array} } & {\begin{array}{*{20}c} {\beta_{{21}} } \\ {\beta_{{31}} } \\ {\begin{array}{*{20}c} {\beta_{{41}} } \\ {0} \\ \end{array} } \\ \end{array} } & {\begin{array}{*{20}c} 0 \\ {\beta_{{23}} } \\ {\begin{array}{*{20}c} 0 \\ {\beta_{{52}} } \\ \end{array} } \\ \end{array} } \\ \end{array} \:\:\:\:\begin{array}{*{20}c} {\beta_{{30}} } & {\beta_{{40}} } & {0} \\ {\beta_{{31}} } & {\beta_{{41}} } & {0} \\ {\begin{array}{*{20}c} {\beta_{{23}} } \\ {0} \\ {\begin{array}{*{20}c} 0 \\ {\beta_{{53}} } \\ \end{array} } \\ \end{array} } & {\begin{array}{*{20}c} 0 \\ {0} \\ {\begin{array}{*{20}c} 0 \\ {\beta_{{54}} } \\ \end{array} } \\ \end{array} } & {\begin{array}{*{20}c} {\beta_{{52}} } \\ {\beta_{{53}} } \\ {\begin{array}{*{20}c} {\beta_{{54}} } \\ {0} \\ \end{array} } \\ \end{array} } \\ \end{array} } \right] \\ & - \left[ {\begin{array}{*{20}c} 0 & {0} & {\eta \:} \\ {0} & {0} & {0} \\ {\begin{array}{*{20}c} {\eta \:} \\ {0} \\ {\begin{array}{*{20}c} 0 \\ {0} \\ \end{array} } \\ \end{array} } & {\begin{array}{*{20}c} 0 \\ {0} \\ {\begin{array}{*{20}c} 0 \\ {0} \\ \end{array} } \\ \end{array} } & {\begin{array}{*{20}c} 0 \\ {\:0} \\ {\begin{array}{*{20}c} 0 \\ {0} \\ \end{array} } \\ \end{array} } \\ \end{array} \:\:\:\:\begin{array}{*{20}c} 0 & {0} & {0} \\ {0} & {0} & {0} \\ {\begin{array}{*{20}c} 0 \\ {\:0} \\ {\begin{array}{*{20}c} 0 \\ {0} \\ \end{array} } \\ \end{array} } & {\begin{array}{*{20}c} 0 \\ {0} \\ {\begin{array}{*{20}c} 0 \\ {0} \\ \end{array} } \\ \end{array} } & {\begin{array}{*{20}c} 0 \\ {\:0} \\ {\begin{array}{*{20}c} 0 \\ {0} \\ \end{array} } \\ \end{array} } \\ \end{array} } \right] \\ \end{aligned}$$

$\:{T}_{10}$ is the tunneling applied component, and the transition coefficients $\:{\beta\:}_{ij}$ ($\:=\frac{{A}_{ij}}{2}+\frac{1}{{\tau\:}_{t}})$, with $\:{A}_{ij}(=\frac{{{\mu\:}_{ij}}^{2}{\omega\:}_{ij}^{2}}{3\pi\:\hslash\:{\epsilon\:}_{s}{c}^{3}})$ is the Einstein coefficient, $\:{{\uptau\:}}_{t}$ is the dipole dephasing time,$\:\:{\epsilon\:}_{s}$ is the semiconductor QD dielectric constant, $\:{\omega\:}_{ij}$ is the transition frequency between QD $\:\left| i \right\rangle$ and $\:\left| j \right\rangle$ states, and $\:{\mu\:}_{ij}$ is the transition momentum, which is here either for QD-QD or WL-QD transition (OPW is used in calculating the latter). The momentum calculation is explained in^12^. $\:\eta\:$ is defined after that with the Rabi field.

### The density matrix equations of the DQD-MNP system

**Fig. 2:**
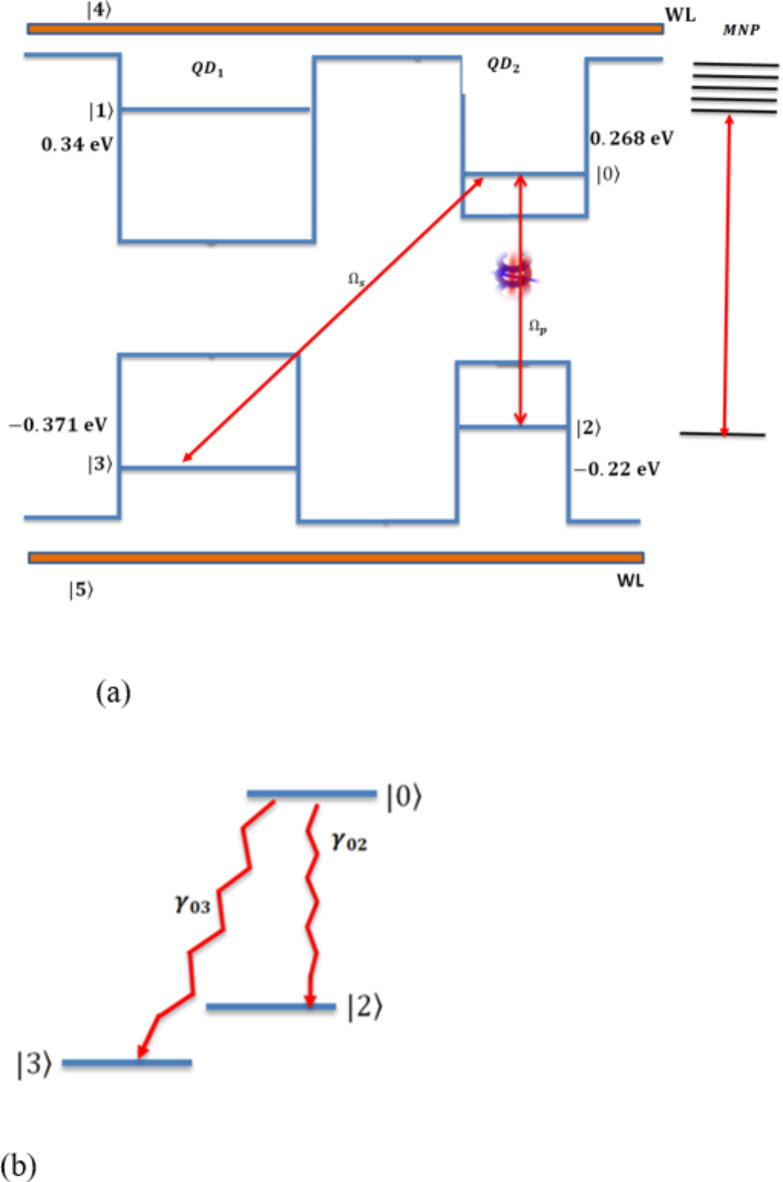
DQD-MNP system: (**a**) DQD-MNP under the generated and OAM probe fields, (**b**) the SGC contribution.

From the density matrix theory, the density operators using the rotating wave approximation for the system in Fig. 2, under the OAM optical probe field $\:{\varOmega\:}_{p}$ and the generated field $\:{\varOmega\:}_{s}$ considering the SGC term, are written as,

3 $$\begin{aligned} \:\mathop {\rho \:}\limits^{.} _{{20}} & = - [i\Delta \:_{{20}} + \left( {\gamma \:_{0} + \gamma \:_{2} } \right)]\rho \:_{{20}} + \left[ {i\Omega \:_{p} \left( {\rho \:_{{00}} - \rho \:_{{22}} } \right)} \right] + \left( {i\beta \:_{{21}} \:\rho \:_{{10}} } \right) \\ & + \left( {i\:sgc\:\rho \:_{{30}} } \right) - \left( {iT_{{10}} \rho \:_{{21}} } \right) - \left( {i\Omega \:_{s} \:\rho \:_{{23}} } \right) - \left( {i\beta \:_{{40}} \:\rho \:_{{24}} } \right) - \left( {i\beta \:_{{52}} \:\rho \:_{{25}} } \right) \\ \end{aligned}$$

4 $$\begin{aligned} \:\mathop {\rho \:}\limits^{.} _{{30}} = & - [\left( {\gamma \:_{0} + \gamma \:_{3} } \right)]\rho \:_{{30}} + \left[ {i\Omega \:_{s} \left( {\rho \:_{{00}} - \rho \:_{{33}} } \right)} \right] + \left( {i\beta \:_{{31}} \:\rho \:_{{10}} \:} \right) + \:\left( {i\:sgc\:\rho \:_{{20}} } \right) \\ & - \left( {iT_{{10}} \rho \:_{{31}} } \right) - \left( {i\Omega \:_{p} \:\rho \:_{{32}} } \right) - \left( {i\beta \:_{{35}} \:\rho \:_{{50}} } \right) - \left( {i\beta \:_{{40}} \:\rho \:_{{34}} } \right) \\ \end{aligned}$$

With $$\:{\gamma\:}_{i}$$ is the relaxation from the state $$\:\left| i \right\rangle$$, $$\:{\varDelta\:}_{20}$$ is the detuning under the applied optical probe field, $$\:sgc$$$$\:(=\text{p}\sqrt{{\gamma\:}_{02}{\gamma\:}_{03}})$$ is the spontaneously generated coherence. With $$\:{\gamma\:}_{ij}$$ is the relaxation between states $$\:\left| i \right\rangle$$ and $$\:\left| j \right\rangle$$. The cross-coupling SGC parameter p is defined by

5 $$\:\text{p}=\frac{{\overrightarrow{\mu\:}}_{20}.{\overrightarrow{\mu\:}}_{30}}{\left|{\overrightarrow{\mu\:}}_{20}.{\overrightarrow{\mu\:}}_{30}\right|}=\text{cos}\theta$$

This definition is similar to that in the literature^15,16^.

As long as the only considered fields are $\:{\varOmega\:}_{p}$ and $\:{\varOmega\:}_{s}$, the terms in the density matrix with terms containing $\:{\beta\:}_{ij}$ are neglected in $\:{\rho\:}_{21}$, $\:{\rho\:}_{43}$, $\:{\rho\:}_{23}$. Then, from the density matrix relations, one can use that $\:{\rho\:}_{21}=\frac{-\left(i{T}_{10}{\rho\:}_{20}\right)}{\left({\gamma\:}_{2}+{\gamma\:}_{1}\right)}$, $\:{\rho\:}_{43}=\frac{-\left(i{T}_{10}{\rho\:}_{20}\right)}{\left({\gamma\:}_{4}+{\gamma\:}_{3}\right)}$, $\:{\rho\:}_{23}=\frac{-\left(i{\varOmega\:}_{p}{\rho\:}_{03}\right)}{\left({\gamma\:}_{2}+{\gamma\:}_{3}\right)}$. Using these relations into $\:{\rho\:}_{30}$, gives,

$$\:{\rho\:}_{30}=\left[\frac{\left({\gamma\:}_{0}+{\gamma\:}_{3}\right){(\gamma\:}_{2}+{\gamma\:}_{3})}{\left({\gamma\:}_{0}+{\gamma\:}_{3}\right){(\gamma\:}_{2}+{\gamma\:}_{3}\left)\right)-{{\varOmega\:}^{2}}_{p}}\right]*\left[\frac{{i\varOmega\:}_{s}}{\left({\gamma\:}_{0}+{\gamma\:}_{3}\right)}+\frac{{\rho\:}_{20}}{\left({\gamma\:}_{0}+{\gamma\:}_{3}\right){(\gamma\:}_{4}+{\gamma\:}_{3})}*\left\{i\right.*{(\gamma\:}_{4}+{\gamma\:}_{3})*sgc-{(\beta\:}_{40}{T}_{10})\}\right]$$

Neglecting the second-order dependence on the applied field. Here, $\:{{\Omega\:}}_{p}^{2}$ is small as $\:{\varOmega\:}_{p}$ is the weak probe field. Then $\:{\rho\:}_{03}$ becomes,

6 $$\:{\rho\:}_{30}=\left[\frac{{i\varOmega\:}_{s}}{\left({\gamma\:}_{^\circ\:}+{\gamma\:}_{3}\right)}+\left\{\left(isgc\right)*\left({\gamma\:}_{4}+{\gamma\:}_{3}\right)-\left({\beta\:}_{40}{T}_{10}\right)\right\}\frac{{\rho\:}_{20}}{{A}_{d30}}\right]$$

Where $\:{A}_{d30}=\left({\gamma\:}_{0}+{\gamma\:}_{3}\right)\left({\gamma\:}_{4}+{\gamma\:}_{3}\right)$. Also, denote, $\:{A}_{z20}=\left[\frac{{(\gamma\:}_{1}+{\gamma\:}_{2}\left)\right[{i\varDelta\:}_{20}+\left({\gamma\:}_{0}+{\gamma\:}_{2}\right)]}{{{T}_{10}^{2}+\left[\right(\gamma\:}_{1}+{\gamma\:}_{2}\left)\right({i\varDelta\:}_{20}+\left({\gamma\:}_{0}+{\gamma\:}_{2}\right)\left)\right]}\right]$, $\:{A}_{d20}$*=*$\:\frac{1}{{i\varDelta\:}_{20}+\left({\gamma\:}_{0}+{\gamma\:}_{2}\right)}$, then,

$$\:{\rho\:}_{20}={A}_{z20}{A}_{d20}[{i\varOmega\:}_{20}+(\left(i*sgc\right)-\frac{{\varOmega\:}_{s}{\varOmega\:}_{p}}{\left({\gamma\:}_{2}+{\gamma\:}_{3}\right)}\left){\rho\:}_{30}\right]$$,

Now, substitute $\:{\rho\:}_{30}$ into $\:{\rho\:}_{20}$ relation to get,

$$\begin{aligned} \:\rho \:_{{20}} *\left\{ {1 - A_{{z20}} A_{{d20}} \left( {isgc - \frac{{\Omega \:_{s} \Omega \:_{p} }}{{\gamma \:_{0} + \gamma \:_{3} }}} \right)*\frac{1}{{A_{{d30}} }}\{ \left( {i\:sgc} \right)\left( {\gamma \:_{4} + \gamma \:_{3} } \right) - (\beta \:_{{40}} T_{{10}} )} \right\} & = A_{{z20}} A_{{d20}} \left( {i\Omega \:_{p} } \right) \\ & + A_{{z20}} A_{{d20}} \left( {\frac{{i\Omega \:_{s} }}{{\gamma \:_{0} + \gamma \:_{3} }}} \right)*\left( {i\:sgc} \right) - \left( {\frac{{\Omega \:_{s} \Omega \:_{p} }}{{\gamma \:_{0} + \gamma \:_{3} }}} \right)\left( {\frac{{i\Omega \:_{s} }}{{\gamma \:_{0} + \gamma \:_{3} }}} \right)A_{{d20}} A_{{z20}} \\ \end{aligned}$$

Neglecting the last term (taking only the linear dependence on the applied fields. Denote,

$$\:{Add}_{20}=[1-{A}_{z20}{A}_{d20}\left(i\:sgc\right)*\frac{1}{{A}_{d30}}\left\{\left(i\:sgc\right)\left({\gamma\:}_{4}+{\gamma\:}_{3}\right)-{(\beta\:}_{40}{T}_{10}\right)\}]$$

The density matrices in Eqs. (1), (2) can be written as,

$$\:{\rho\:}_{20}=\left\{{A}_{z20}{A}_{d20}\left({i\varOmega\:}_{p}\right)+{A}_{z20}{A}_{d20}\left(\frac{i{\varOmega\:}_{s}}{{\gamma\:}_{0}+{\gamma\:}_{3}}\right)*\left(i\:sgc\right)\right\}/{Add}_{20}$$

Or,

7 $$\:{\rho\:}_{20}={a}_{1}{\varOmega\:}_{p}+{{b}_{1}\varOmega\:}_{s}$$

With

8 $$\:{a}_{1}=\frac{i{A}_{z20}{A}_{d20}}{{Add}_{20}}$$

9 $$\:{b}_{1}=\frac{-{A}_{z20}{A}_{d20}sgc}{{{(\gamma\:}_{0}+{\gamma\:}_{3})Add}_{20}}$$

To get the relation of $\:{\rho\:}_{30}$, denote,

$$\:{A}_{a30}=\left\{\left(i\:sgc\right)*\left({\gamma\:}_{4}+{\gamma\:}_{3}\right)-{(\beta\:}_{40}{T}_{10}\right)\}$$

The relation of $\:{\rho\:}_{30}$ can be written as,

$$\:{\rho\:}_{30}=\left[\frac{{i\varOmega\:}_{s}}{\left({\gamma\:}_{^\circ\:}+{\gamma\:}_{3}\right)}+{A}_{a30}\left({a}_{1}{{\Omega\:}}_{p}+{b}_{1}{{\Omega\:}}_{s}\right)*\frac{1}{{A}_{d30}}\right]$$

Then,

$$\:{\rho\:}_{30}=\frac{{\left[i\left({\gamma\:}_{4}+{\gamma\:}_{3}\right)+{A}_{a30}{b}_{1}\right]\varOmega\:}_{s}}{{A}_{d30}}+\frac{{{A}_{a30}{a}_{1}\varOmega\:}_{p}}{{A}_{d30}}$$

The second relation is

10 $$\:{\rho\:}_{30}={a}_{2}{\varOmega\:}_{p}+{b}_{2}{\varOmega\:}_{s}$$

11 $$\:{b}_{2}=\frac{{\left[i\left({\gamma\:}_{4}+{\gamma\:}_{3}\right)+{A}_{a30}{b}_{1}\right]}_{\:}}{{A}_{d30}}$$

12 $$\:{a}_{2}=\frac{{A}_{a30}{a}_{1}}{{A}_{d30}}$$

### The Rabi fields

The following relations are derived for the generated and probe Rabi fields,

13 $$\:{{\Omega\:}}_{\text{s}}\left(z\right)=\frac{{a}_{1}{b}_{2}}{\left[{a}_{1}{b}_{2}{-b}_{1}{a}_{2}\right]}{{\Omega\:}}_{\text{P}}\left(0\right)\left[{e}^{i{A}_{s}{b}_{2}z}-1\right]+\frac{{a}_{1}{b}_{2}}{\left[{a}_{1}{b}_{2}{-a}_{2}{b}_{1}\right]}i{{\Omega\:}}_{\text{P}}\left(0\right)\left[\frac{{a}_{1}}{{b}_{2}}\text{sin}\left({A}_{s}{b}_{2}z\right)\frac{{A}_{P}}{{A}_{s}}-\frac{{b}_{1}{a}_{2}}{{a}_{1}^{2}}\frac{{A}_{s}}{{A}_{P}}i\:\text{s}\text{i}\text{n}\left({A}_{P}{a}_{1}z\right)\right]$$

14 $$\:{\varOmega\:}_{P}\left(z\right)={\varOmega\:}_{P}\left(0\right)+{\varOmega\:}_{P}\left(0\right)\frac{{b}_{1}{a}_{2}}{{a}_{1}^{2}}\frac{{A}_{s}}{{A}_{P}}i\text{s}\text{i}\text{n}\left({A}_{P}{a}_{1}z\right)-\frac{{b}_{1}}{{a}_{1}}{\varOmega\:}_{s}\left(z\right)$$

Their derivation is set in Appendix A. Note that the vortex defines the OAM probe optical field is^18^,

15 $$\Omega _{p} \left( {z = 0} \right) = \Omega _{{p0}} ~(\frac{r}{w})^{{\left| l \right|}} ~e^{{ - \frac{{r^{2} }}{{w^{2} }}}} e^{{il\emptyset }}$$

Where $\:r$ is the distance from the vortex, $\:w$ is the beam waist, $\:l$ is the OAM number along the propagation direction and $\:\varnothing\:$ is the azimuthal angle. Note that $\:{{\Omega\:}}_{p0}$ is the applied probe field defined as^19^,

16–a $$\:{{\Omega\:}}_{p0}={\varOmega\:}_{p0\:}^{\left(1\right)}-\eta$$

The above definition is at strong coupling, and,

16–b $$\:{{\varOmega\:}_{p0\:}^{\left(1\right)}=\varOmega\:}_{p0\:}^{\left(0\right)}\left(1+\frac{{S}_{a\:}\gamma\:{a}^{3}}{{R}^{3}}\right)$$

With the applied probe Rabi frequency^19^,

16–c $$\Omega _{{p0}}^{{(0)}} = \frac{{\mu _{{02}} E_{{02}}^{{(0)}} }}{{2\hbar \varepsilon _{{effs}} }}$$

where $\:{\mu\:}_{20}$ is the momentum of the transition between the states $\:\left| 0 \right\rangle$, $\:\left| 2 \right\rangle$ and $\:{E}_{20}$ is the strength of the probe field applied between these states. The Rabi frequency at weak coupling is defined by $\:{{\Omega\:}}_{\text{p}0}^{\left(0\right)}$ alone. The calculated momentum $\:{\mu\:}_{20}$ and also WL-QD momenta is set in the Appendix B, and,

16–d $$\:\eta\:=\frac{{S}_{a}^{2}\gamma\:{\mu\:}_{20}^{2}}{\hslash\:{\epsilon\:}_{effs}^{2}}\frac{{a}^{3}}{{R}^{6}}$$

Note that $\:{S}_{a}$ is the electric field direction where $\:{S}_{a}=2$ is for the z-axis polarization and $\:{S}_{a}=-1$ is for the parallel direction. The screened dielectric constant in the DQD or the MNP is defined by^19^,

16–e $$\:{\epsilon\:}_{effi}=\frac{({2\epsilon\:}_{0}+{\epsilon\:}_{i})}{{3\epsilon\:}_{0}}$$

where $\:{\epsilon\:}_{i}$ is either $\:{\epsilon\:}_{s}$ for the semiconductor DQD or $\:{\epsilon\:}_{m}$ for the MNP. Also, define

16–f $$\:\gamma\:=\frac{{{\upvarepsilon\:}}_{m\:}\left(w\right)-{\epsilon\:}_{0}}{{{\upvarepsilon\:}}_{m\:}\left(w\right)+2{\epsilon\:}_{0}}$$

Equation (13) defines the strong coupling^11^. The dielectric function of the MNP is defined by^20^,

17–a $$\:{\epsilon\:}_{m}\left(w\right)={\epsilon\:}_{d}\left(w\right)+{\epsilon\:}_{s}\left(w\right)$$

where $\:{\epsilon\:}_{s}\left(w\right)$ represents the contribution of the s-state electrons to the dielectric constant. It is defined in the Drude model by^20^,

17–b $$\:{\epsilon\:}_{s}\left(w\right)=1-\frac{{w}_{p}^{2}}{{w}^{2}+iw\left({\gamma\:}_{bulk}+\frac{A{v}_{f}}{{r}_{m}}\right)}$$

where $\:{\gamma\:}_{bulk}$ is the damping constant of the bulk metal, $\:{v}_{f}$ is the electron velocity at Fermi energy, $\:{w}_{p}$ is the plasma frequency of the metal, and $\:A$ is related to the MNP electron scattering. In Eq. (14–a), $\:{\epsilon\:}_{d}\left(w\right)$ is the contribution of the d-state electrons in the MNP. For the gold MNP at the plasma frequency $\:{w}_{p}=2.5\:\left(eV\right)$, it defined by^20^,

17–c $$\:{\epsilon\:}_{d}\left(w\right)=1.15+i10.5$$

Using the Drude model in the strong coupling QD-MNP is a common assumption. This results from the high concentration of electrons, as well as in MNP^21^.

## Results and discussion

This work simulates the optical properties of the DQD-MNP structure subjected to a probe optical field with OAM considering SGC in the structure. While the pump and OAM probe field relations are derived analytically, the DQD energy states and the WL-QD and QD-QD transition momenta ($\:{\mu\:}_{ij}$) are calculated via MAOUD-37 software written under MATLAB^12^. So, the work here differs from other works regarding material property. Such property comes from calculating QD energy states and momenta for the structure under study. The WL state with WL-QD transition momenta are also calculated in the computation, considering the inevitable OPW between WL and QDs. This is not considered earlier. The parameters used in the calculations are listed in Table 1. The calculated momenta are also listed to make it easy to repeat calculations. Note that the relaxations $\:{\gamma\:}_{02},\:{\gamma\:}_{03}$ that form the two parts of the SGC, Fig. 2, are taken as $\:{\gamma\:}_{02}=30{\gamma\:}_{0}$ and $\:{\gamma\:}_{03}=2000{\gamma\:}_{0}$. Such difference takes into account their difference in energy; in addition, the state $\:\left| 3 \right\rangle$ is in another QD. Then $\:{\gamma\:}_{2}={\gamma\:}_{02}$, and $\:{\gamma\:}_{3}={\gamma\:}_{03}$ are taken and $\:{\gamma\:}_{1}={\gamma\:}_{0}$. For the WL state, the relaxation is taken at longer time than the QD,$\:\:{\gamma\:}_{4}=1/\left(1ns\right)$^22^. The cross-coupling SGC parameter p values are chosen between $\:0$ and $\:1$ as in the literature^15,16^.

Unless stated otherwise, the following data are used: $\:\text{a}=16\:nm$, $\:{r}_{m}=6\:nm$, $\:\text{p}=0.1$, device length $\:L=200\:\mu\:m$, beam wais $\:w=20\:\mu\:m$, the distance from the vortex$\:\:r=2w$, the OAM number along the propagation direction$\:\:l$=1 and the azimuthal angle $\:\varnothing\:=0.1$, and $\:{A}_{P}={A}_{s}=40$. Such data are chosen depending on what is used in the literature, such as in^18^.

**Table 1:** The parameters used in the calculations.

Parameter	Symbol	Value (unit)	Ref.	
The QD relaxation rates	$\:{\gamma\:}_{0}$	$\:1/\left(0.4\:ns\right)$	^22^	
The WL relaxation rates	$\:{\gamma\:}_{4},\:{\gamma\:}_{5}$	$\:1/\left(1\:ns\right)$	^22^	
Dephasing time	$\:{{\uptau\:}}_{t}$	$\:500\:ps$	^23^	
InAs QD dielectric constant	$\:{{\upepsilon\:}}_{\text{s}}$	$\:15.15{\epsilon\:}_{0}$	^24^	
Damping constant of the bulk metal	$\:{\gamma\:}_{bulk}$	$\:0.1\:\left(eV\right)$	^20^	
Electron velocity at Fermi energy	$\:{v}_{f}$	$\:1.4\times\:{10}^{6}\:m/sec$	^20^	
Plasma frequency of the metal	$\:{w}_{p}$	$\:2.5\:\left(eV\right)$	^20^	
Tunneling component	$\:{T}_{10}$	$\:{900\gamma\:}_{0}$		
Input probe frequency (considering screened metal contribution)	$\:{{\Omega\:}}_{p0}$	$\:6.35\times\:{10}^{5}$		

Calculated QD momenta				
Momentum	Value (nm.e)	Momentum		Value (nm.e)
$\:{\mu\:}_{10}$	11.2635	$\:{\mu\:}_{25}$		6.2047
$\:{\mu\:}_{20}$	0.0161	$\:{\mu\:}_{35}$		6.1059
$\:{\mu\:}_{03}$	0.0055	$\:{\mu\:}_{14}$		1.4652
$\:{\mu\:}_{32}$	13.4620	$\:{\mu\:}_{04}$		1.3725
$\:{\mu\:}_{31}$	0.0047	$\:{\mu\:}_{21}$		0.0139

**Fig. 3:**
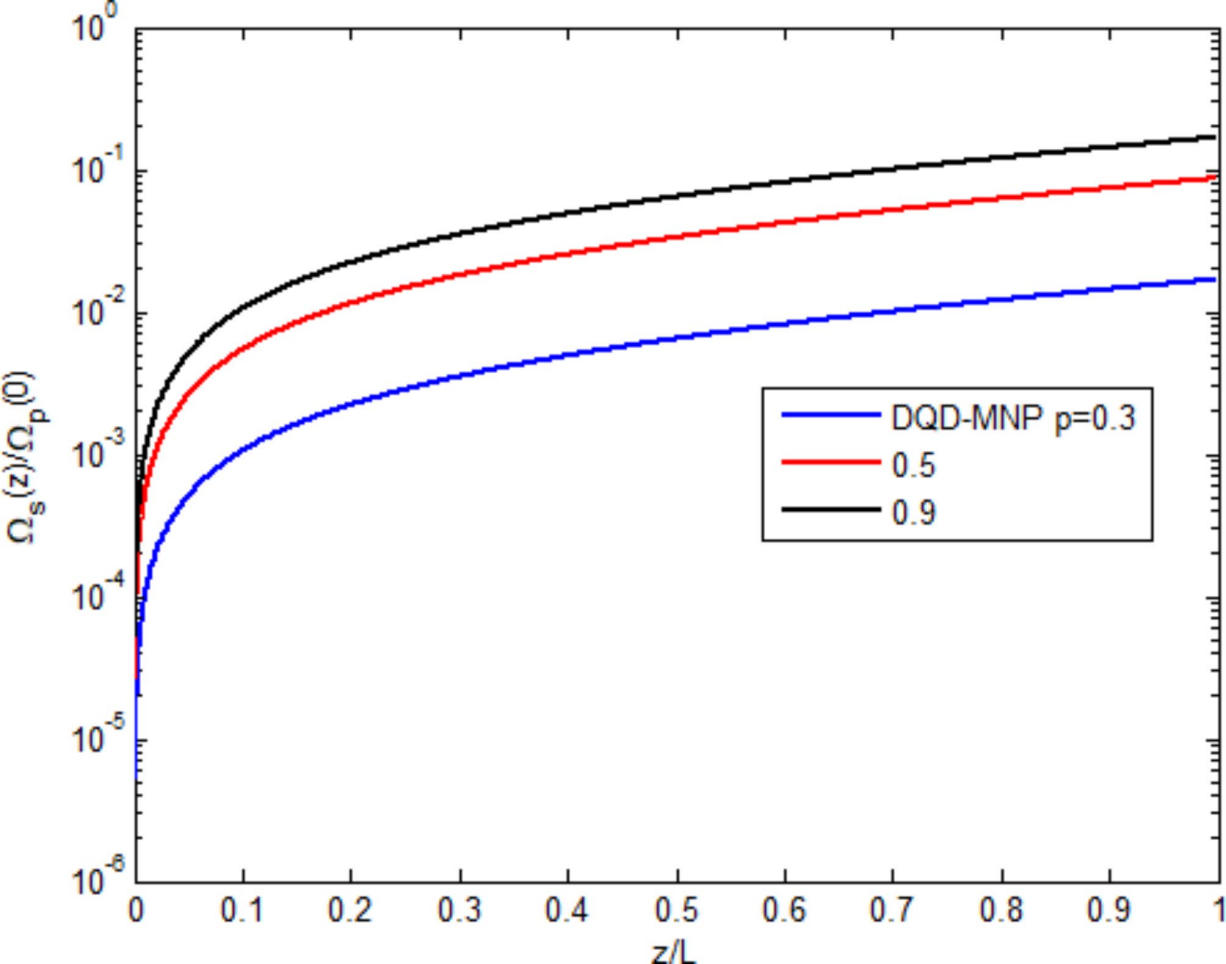
Normalized generated field versus normalized distance at three SGC ratios.

Figure 3 shows the field-generated $\:{{\Omega\:}}_{s}\left(z\right)$ normalized to the applied probe field $\:{{\Omega\:}}_{p}\left(0\right)$ as a function of the normalized spatial distance $\:(z/L)$ under three spontaneously generated coherence (SGC) ratios $\:(\text{p}=0.3,\:0.5,\:0.9)$. The generated field begins from zero and linearly increases with distance. The highest value of the generated field is $\:\sim0.2\:{{\Omega\:}}_{p}\left(0\right)$ at $\:(p=0.9)$. Such limitation of the field generated can be ascribed to the absorption through the system. The generated field ratio increases with SGC, showing the importance of SGC in increasing the generated field. The behavior of increasing the generated field along the device length with SGC has already been demonstrated in other works^2,6^. SGC adds extra atomic coherence, causing an EIT, increasing the nonlinearity, and reducing absorption. This causes the raising of curves with increasing the SGC parameter (p)^16,25^.

**Fig. 4:**
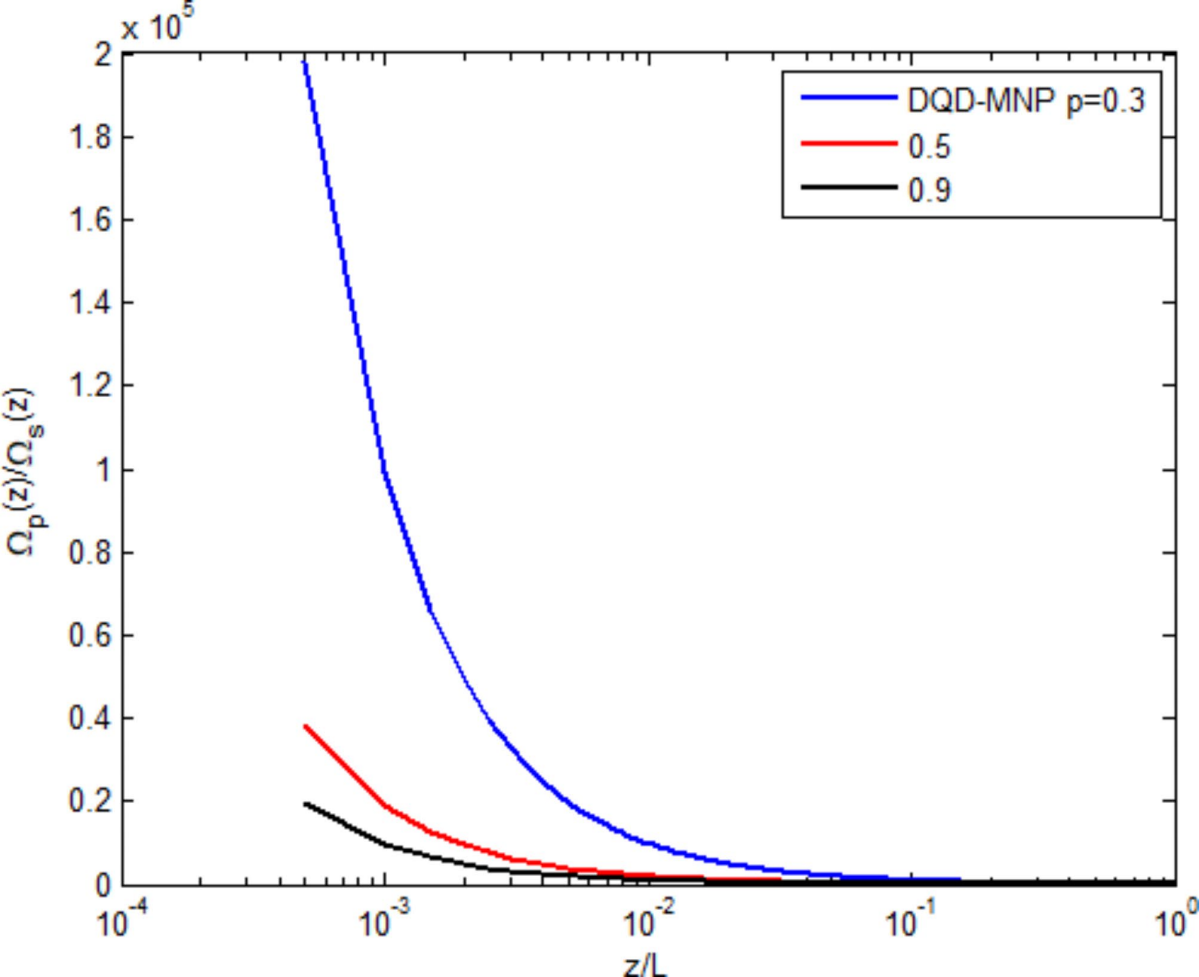
Normalized probe field versus normalized distance at three SGC ratios.

Figure 4 shows the applied probe field ratio $\:{{\Omega\:}}_{p}\left(z\right)/{{\Omega\:}}_{s}\left(z\right)$ as a function of the normalized spatial distance $\:(z/L)$ under the SGC ratios $\:(\text{p}=0.3,\:0.5,\:0.9)$. The applied field linearly descends due to the absorption through the device. The high SGC ratio reduces the applied field via the generating field. Such behavior is also shown in^13^.

**Fig. 5:**
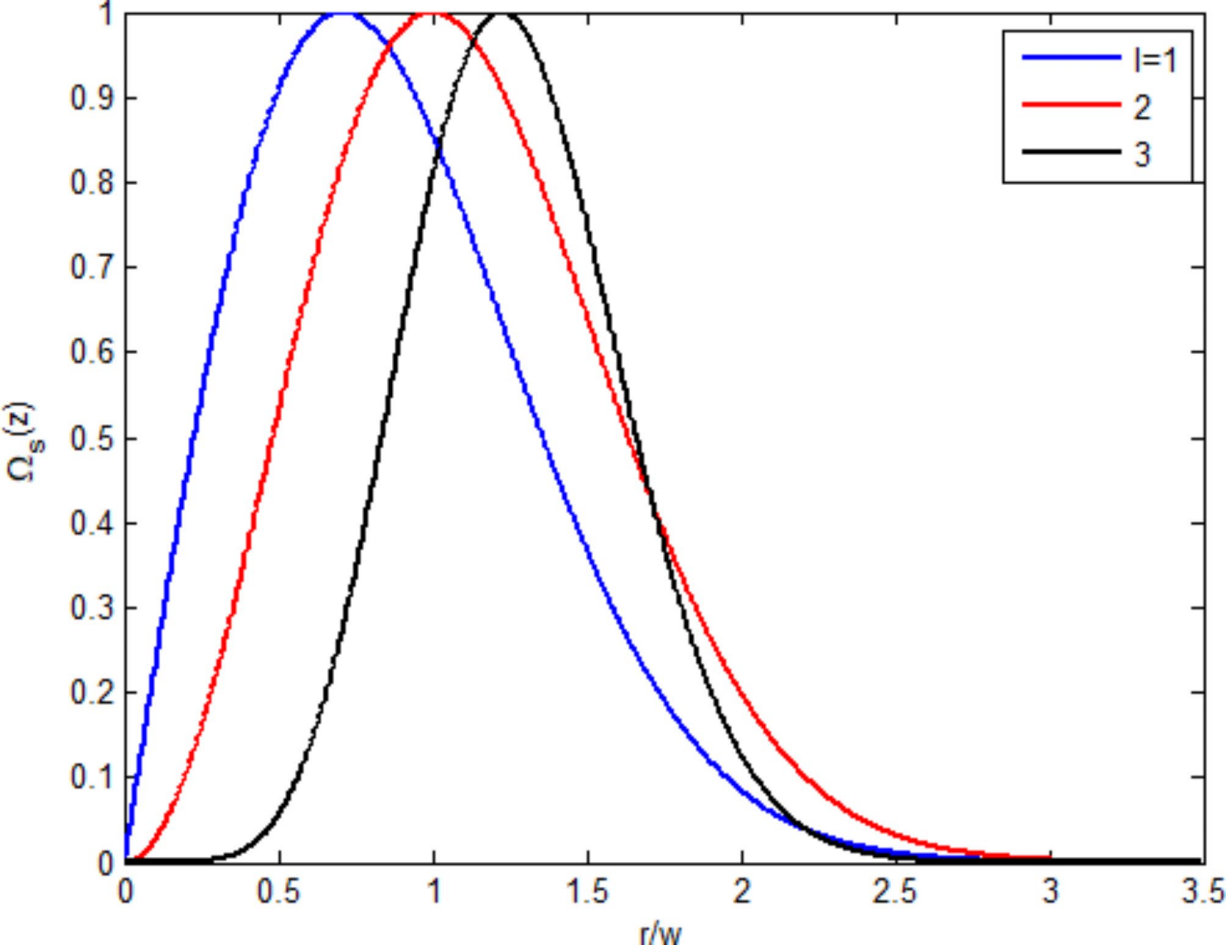
Normalized generated probe field versus normalized distance from the vortex at three OAM numbers.

Figure 5 shows the normalized OAM-generated field concerning distance from the vortex $\:\left(r\right)$ normalized by the beam waist $\:\left(w\right)$ at three OAM numbers. Increasing the OAM number amplifies the generated field and shifts the amplified peak to a long vortex distance. The behavior of moving the peak of the generated field is also shown in^5^. More increments of the OAM number reduce the generated field at long distances, as shown in the black curve.

The main features in the probe and generated fields of the atomic structures under structured light are studied in Figs. 3, 4, 5, and as shown in the above results, the results are not much different from other structures.

**Fig. 6:**
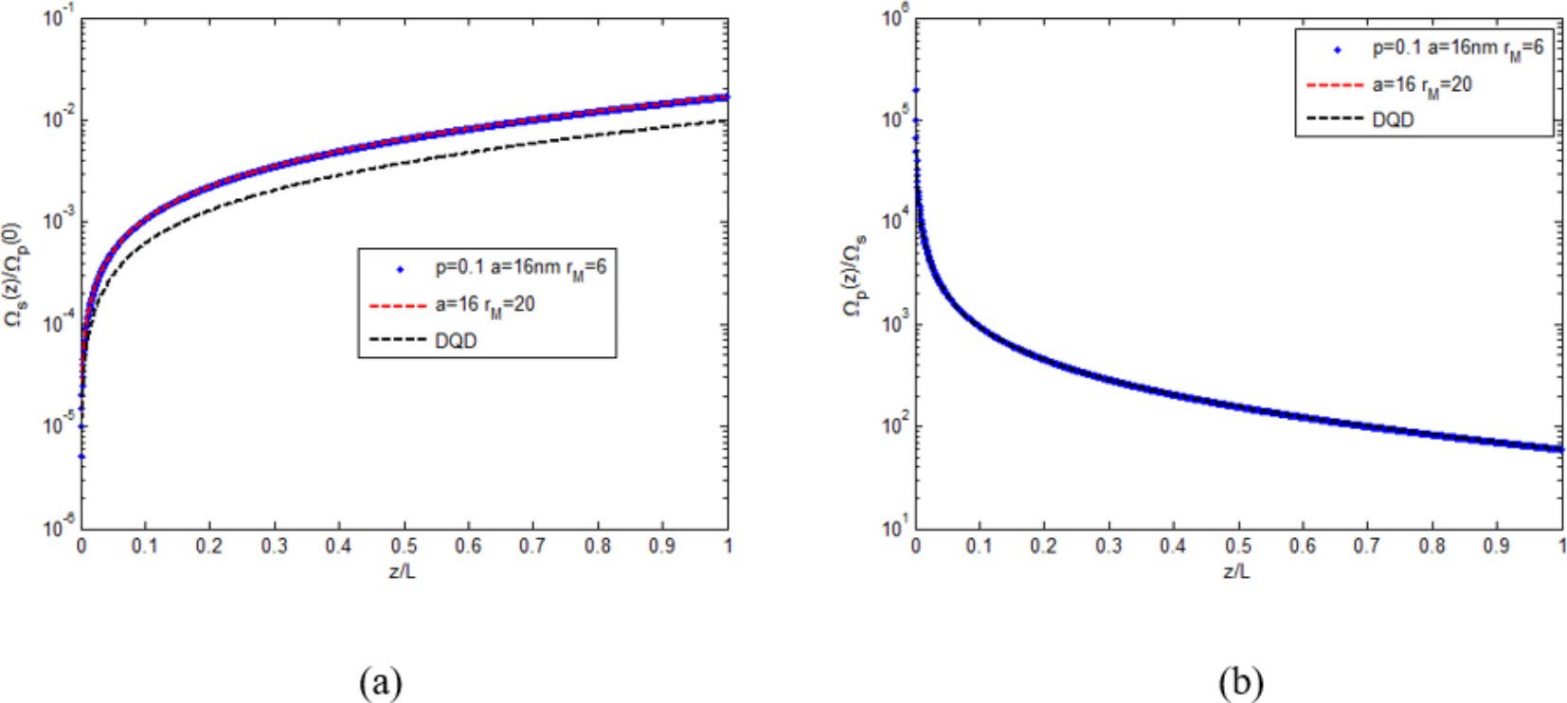
The behavior of the fields along the normalized distance in the DQD-MNP system at strong coupling (blue dotted), DQD-MNP system at weak coupling (red-dotted), and DQD system (black-dotted): (**a**) normalized generated field. (**b**) normalized probe field.

To compare the DQD-MNP structure features with other atomic structures, Fig. 6 is shown. It compares the performance of the DQD-MNP at strong coupling (blue-dotted) with its weak coupling case (red-dashed) and with the DQD (black-dashed curve) systems. Figure 6a compares field generation. For the three systems, the signal field increases with distance. The signal field generation in the DQD-MNP system doubles that of the DQD system alone. Such behavior prefers the DQD-MNP system to the DQD system alone. Figure 6b shows the probe field applied to the systems under study, which is reduced with distance due to the conversion into the generated field.

**Fig. 7:**
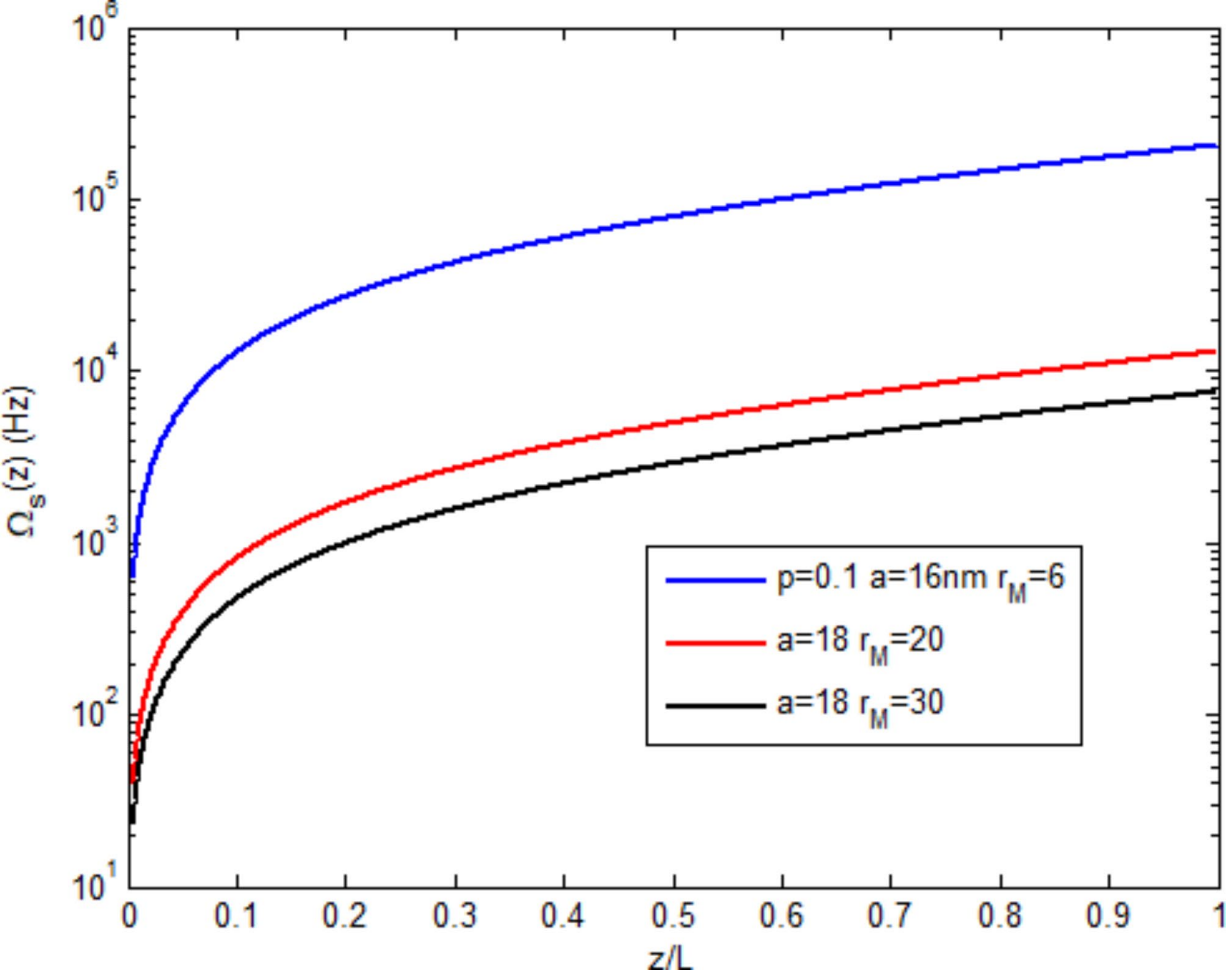
The behavior of the generated field along the normalized distance in the DQD-MNP system at strong coupling (blue) and DQD-MNP system at weak coupling (red and black).

Figure 7 compares the coupling cases of the MNP-DQD system, where the strong coupling case $\:(a=16\:nm,\:{r}_{M}=6\:nm$–blue curve) is compared with two weak coupling cases: the red curve $\:(a=18\:nm,\:{r}_{M}=20\:nm$ ) and black curve $\:(a=18\:nm,\:{r}_{M}=30\:nm)$. The signal field at the strong coupling is higher by more than one order than the weak cases. Increasing MNP-DQD distance ($\:{r}_{M})$ reduces the generated field. Thus, strong coupling is preferred. From Figs. 6, 7, the effect of coupling the MNP to the DQD is evident, where the generated field in the DQD-MNP structure becomes high compared to the DQD structure alone. This can be explained by Eq. (16) above, where parts are added due to the MNP contribution. The QD-MNP system has a strong exciton-plasmon coupling without needing an externally applied pump field or incoherent fields. An energy transfer from the MNP to the QD suggests the generation of quantum coherence in the QD-MNP system^26^. Such coherence reduces the absorption^25^ and increases the generated field.

**Fig. 8:**
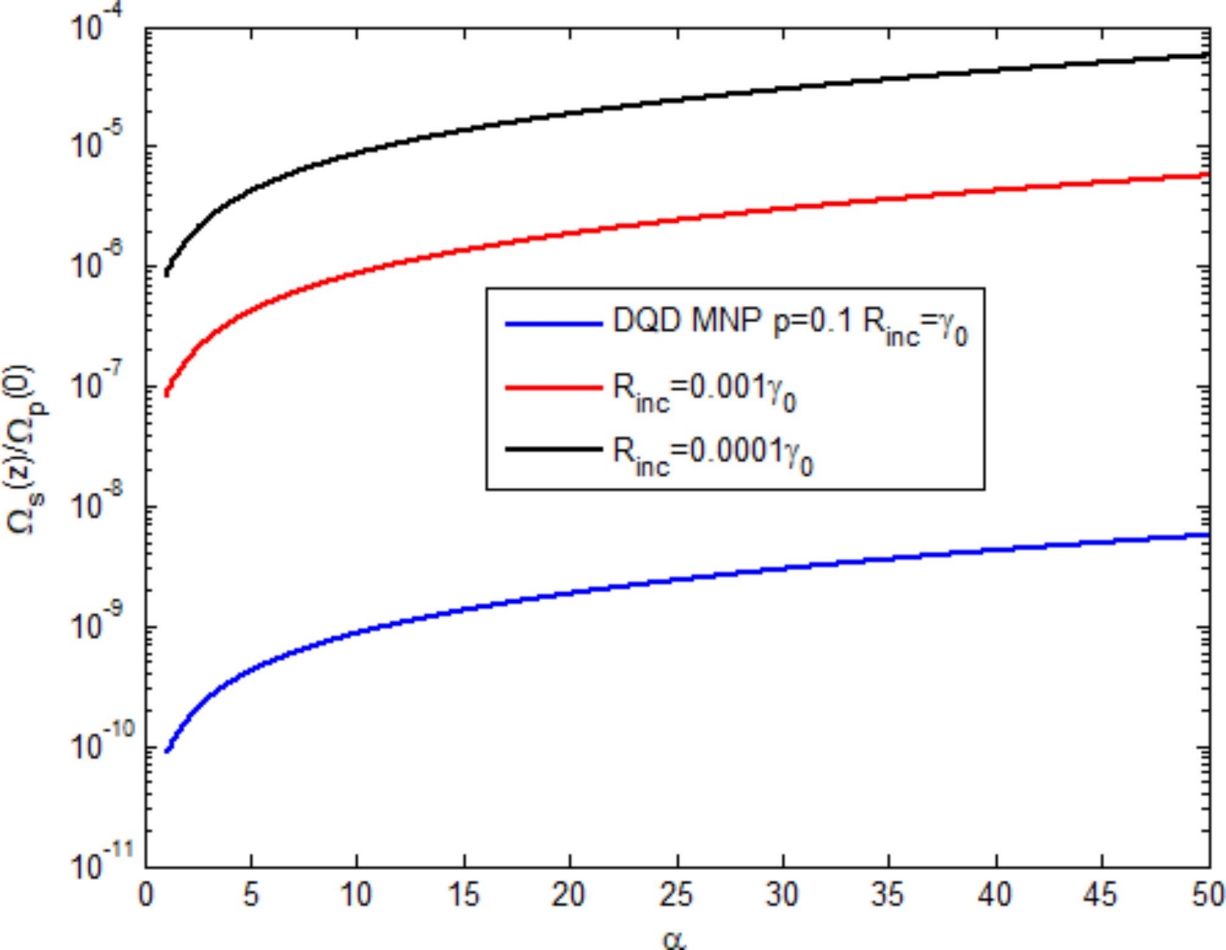
The effect of the optical depth at three incoherent pumpings on the normalized generated field.

Equations (1), (2) are then modified to include the incoherent pumping term, and the formulation is shown in Appendix C. Figure 8 shows the generated field as a function of the optical depth $\:\left(\alpha\:\right)$ at three values of the incoherent pumping $\:\left({R}_{inc}\right)$. It is shown that the incoherent pumping reduces the generated field, a behavior shown in^6^ for a three-level atomic system.

## Conclusions

An analytical model for the OAM probe field and the generated one in the DQD-MNP system is derived. WL-QD transitions, with OPW, in addition to QD-QD, are considered. SGC is considered. The calculations here are of material property through the computation of the QD-QD and WL-QD transition momenta, which are essential for specifying the Rabi frequency. The results show that SGC increases the generated field. The signal field generation in the DQD-MNP system doubles that from the DQD system alone. So, the DQD-MNP system is preferred to the DQD system. The generated field in the DQD-MNP for the strong coupling case is higher than that for the weak coupling. The strength of the exciton-plasmon coupling depends on the distance separating the strong DQD-MNP structure. At strong coupling (short distances), more energy is transferred from the MNP to the DQD, generating a quantum coherence. This coherence reduces the absorption and increases the generating field. Increasing the DQD-MNP distance reduces the generated field. The model is then extended to include the incoherent pumping, and the relations are modified to include this effect. Small incoherent pumping increases the generated signal. This study compared well with the literature, while those comparing the weak with strong coupling are only done in this work for the first as long as the OAM in the MNP-DQD structure is studied here for the first. The results open the way to study quantum entanglement as quantum coherence increases under SGC. Slow light is also a promising application, as the absorption is reduced with strong coupling.

## Data Availability

This article includes all the data generated or analyzed during this study.

## Appendix A: derivation of optical properties of DQD-MNP with OAM

The following relations are used

A–1 $$\frac{{d\Omega _{s} }}{{dz}} = iA_{s} (a_{2} \Omega _{P} + b_{2} \Omega _{s} )$$

With $A_{s} = \frac{{\alpha _{s} \Gamma }}{{2L}}$ and $\:{\alpha\:}_{s}$ is the optical depth of the signal-generated field. Eq. (A–1) is solved by separating its homogenous and particular parts as follows: for the homogenous solution,

A–2a $$\:\frac{d{{\Omega\:}}_{s}}{dz}=i{A}_{s}{b}_{2}{\varOmega\:}_{s}$$

Giving,

A–2b $$\:{{\Omega\:}}_{s}=A{e}^{i{A}_{s}{b}_{2}Z}+B{e}^{-i{A}_{s}{b}_{2}Z}$$

With $\:A$ and $\:B$ are constants to be calculated. For the particular solution,

A–3a $$\:i{A}_{s}{a}_{2}{{\Omega\:}}_{P}+i{A}_{s}{b}_{2}{\varOmega\:}_{s}=0$$

Giving,

A–3b $$\:{{\Omega\:}}_{s}=\frac{-{a}_{2}{{\Omega\:}}_{P}}{{b}_{2}}C$$

With $\:C$ is a constant to be calculated. Summing homogeneous and the particular solution gives,

A–4 $$\:\:{{\Omega\:}}_{s}=A{e}^{i{A}_{s}{b}_{2}Z}+B{e}^{-i{A}_{s}{b}_{2}Z}-\frac{{a}_{2}{{\Omega\:}}_{P}}{{b}_{2}}C$$

Rewrite Eq. (A–1) using Eq. (A–4),

A–5 $$\:\frac{d{{\Omega\:}}_{s}}{dz}=A\left(i{A}_{s}{b}_{2}\right){e}^{i{A}_{s}{b}_{2}Z}-B\left(i{A}_{s}{b}_{2}\right){e}^{-i{A}_{s}{b}_{2}Z}-\frac{{a}_{2}}{{b}_{2}}C\frac{d{{\Omega\:}}_{P}}{dz}$$

So, one must use the second equation,

A–6 $$\:\frac{d{{\Omega\:}}_{p}}{dz}=i{A}_{p}({a}_{1}{{\Omega\:}}_{p}+{b}_{1}{\varOmega\:}_{s})$$

With $A_{p} = \frac{{\alpha _{p} \Gamma }}{{2L}}$ and $\:{\alpha\:}_{p}$ is the optical depth of the probe field. Using (A–6) in Eq. (A–5), and using it in Eq. (A–1), gives,

$$\:A\left(i{A}_{s}{b}_{2}\right){e}^{i{A}_{s}{b}_{2}Z}-B\left(i{A}_{s}{b}_{2}\right){e}^{-i{A}_{s}{b}_{2}Z}-\frac{{a}_{2}C}{{b}_{2}}\:\left[i{A}_{P}{a}_{1}{{\Omega\:}}_{P}+i{A}_{P}{b}_{1}{\varOmega\:}_{s}\right]=i{A}_{s}{a}_{2}{{\Omega\:}}_{P}+i{A}_{s}{b}_{2}{\varOmega\:}_{s}$$

Applying the following conditions

A–7 $$\:{{\Omega\:}}_{s}\left(Z=0\right)=0,\:\:\:\:\:{{\Omega\:}}_{P}\left(Z=0\right)=\:{{\Omega\:}}_{P}\left(0\right)$$

Where the probe field is applied only. This gives,

A–8 $$\:A\left(i{A}_{s}{b}_{2}\right){e}^{i{A}_{s}{b}_{2}Z}-B\left(i{A}_{s}{b}_{2}\right){e}^{-i{A}_{s}{b}_{2}Z}-\frac{{a}_{2}C}{{b}_{2}}\:\left[i{A}_{P}{a}_{1}{\varOmega\:}_{P}\left(0\right)\right]=i{A}_{s}{a}_{2}{{\Omega\:}}_{P}\left(0\right)$$

Now, return to the second equation, Eq. (A–6). In the same way, the complete solution is given as,

A–9 $$\:{{\Omega\:}}_{P}={D}_{1}{e}^{i{A}_{P}{a}_{1}Z}+{D}_{2}{e}^{-i{A}_{P}{a}_{1}Z}-\frac{{b}_{1}{{\Omega\:}}_{s}}{{a}_{1}}\:{C}_{2}$$

and using into (A–6), one obtains,

A–10 $$\:\frac{d{{\Omega\:}}_{P}}{dz}={iD}_{1}\left({A}_{P}{a}_{1}\right){e}^{i{A}_{P}{a}_{1}Z}-{iD}_{2}{\left({A}_{P}{a}_{1}\right)}^{\:}{e}^{-i{A}_{P}{a}_{1}Z}-\frac{{b}_{1}}{{a}_{1}}{C}_{2}\frac{d{{\Omega\:}}_{s}}{dz}$$

Using Eq. (A–10) into Eq. (A–6) gives,

$$\:{D}_{1}\left(i{A}_{P}{a}_{1}\right)-{D}_{2}\left(i{A}_{P}{a}_{1}\right)-\frac{{b}_{1}}{{a}_{1}}{C}_{2}\frac{d{{\Omega\:}}_{s}}{dz}=i{A}_{P}{a}_{1}\left[{D}_{1}+{D}_{2}-\frac{{b}_{1}}{{a}_{1}}{C}_{2}{{\Omega\:}}_{s}\right]+i{A}_{P}{b}_{1}{\varOmega\:}_{s}$$

Then using Eq. (A–2) in the above relation at $\:Z=0$, gives,

$$\:\left(i{A}_{P}{a}_{1}\right)\left[{D}_{1}-{D}_{2}\right]-\frac{{b}_{1}}{{a}_{1}}{C}_{2}\left[i{A}_{s}{a}_{2}{\varOmega\:}_{P}\left(0\right)\right]=i{A}_{P}{a}_{1}\left[{D}_{1}+{D}_{2}\right]$$

Then,

$$\:{D}_{2}=\frac{-{b}_{1}}{{2a}_{1}}{a}_{2}\frac{{A}_{s}}{{A}_{P}}{\varOmega\:}_{P}\left(0\right){C}_{2}$$

A–11 $$\:\therefore\:\:{\varOmega\:}_{P}\left(z\right){=D}_{1}{e}^{i{A}_{P}{a}_{1}Z}+-\frac{{b}_{1}{a}_{2}}{2{a}_{1}^{2}}\frac{{A}_{s}}{{A}_{P}}{\varOmega\:}_{P}\left(0\right){C}_{2}{e}^{-i{A}_{P}{a}_{1}Z}-\frac{{b}_{1}}{{a}_{1}}{\varOmega\:}_{s}\left(z\right){C}_{2}$$

To get $\:{C}_{2}$, one must use the particular solution from Eq. (A–9), $\:{\varOmega\:}_{P}=-\frac{{b}_{1}}{{a}_{1}}{\varOmega\:}_{s}{C}_{2}$, and substitute it into the particular equation $\:\{0=i{A}_{p}\left({a}_{1}{{\Omega\:}}_{p}+{b}_{1}{\varOmega\:}_{s}\right)\}$ obtained from Eq. (A–6) to get

$$\:i{A}_{P}{a}_{1}\left(-\frac{{b}_{1}}{{a}_{1}}{\varOmega\:}_{s}{C}_{2}\right)+i{A}_{P}{b}_{1}{\varOmega\:}_{s}=0$$

$$\:\therefore\:{C}_{2}=1\:\:\:$$

Substitute into Eq. (A–11) gives,

$$\:{\varOmega\:}_{P}\left(z\right){=D}_{1}{e}^{i{A}_{P}{a}_{1}Z}-\frac{{b}_{1}}{{a}_{1}}\left[\frac{{a}_{2}}{{2a}_{1}}\frac{{A}_{s}}{{A}_{P}}{\varOmega\:}_{P}\left(0\right){e}^{-i{A}_{P}{a}_{1}Z}+{\varOmega\:}_{s}\left(z\right)\right]$$

Applying the boundary conditions (A–7) gives,

A–12 $$\:{D}_{1}={\varOmega\:}_{P}\left(0\right)\left[1+\frac{{b}_{1}{a}_{2}}{2{a}_{1}^{2}}\frac{{A}_{s}}{{A}_{P}}\right]$$

This returns to

A–13 $$\:{\varOmega\:}_{P}\left(z\right)={\varOmega\:}_{P}\left(0\right)+{\varOmega\:}_{P}\left(0\right)\frac{{b}_{1}{a}_{2}}{{a}_{1}^{2}}\frac{{A}_{s}}{{A}_{P}}i\text{s}\text{i}\text{n}\left({A}_{P}{a}_{1}z\right)-\frac{{b}_{1}}{{a}_{1}}{\varOmega\:}_{s}\left(z\right)$$

The same way of obtaining $\:{C}_{2}$ is used to obtain $\:C,$ where it gives $\:C=1$. Now, to get the constants $\:A,B$ one must write Eq. (A–4) in the case $\:z=0,$

$$\:{\varOmega\:}_{s}\left(0\right)=A+B-\frac{{a}_{2}}{{b}_{2}}{\varOmega\:}_{P}\left(0\right)$$

A–14 $$\:A=\frac{{a}_{2}}{{b}_{2}}{\varOmega\:}_{P}\left(0\right)-B$$

From Eq. (A–8), one can write

$$\:i{A}_{s}{a}_{2}{\varOmega\:}_{P}\left(0\right)=A\left(i{A}_{s}{b}_{2}\right)+B\left(i{A}_{s}{b}_{2}\right)-\frac{{a}_{2}{a}_{1}}{{b}_{2}}i{A}_{P}{\varOmega\:}_{P}\left(0\right)$$

Using Eq. (A–14) to get,

$$\:i{A}_{s}{a}_{2}{\varOmega\:}_{P}\left(0\right)=\left(\frac{{a}_{2}}{{b}_{2}}{\varOmega\:}_{P}\left(0\right)-B\right)\left(i{A}_{s}{b}_{2}\right)-B\left(i{A}_{s}{b}_{2}\right)-\frac{{a}_{2}{a}_{1}}{{b}_{2}}i{A}_{P}{\varOmega\:}_{P}\left(0\right)$$

Which gives,

$$\:B=-\frac{{a}_{2}{a}_{1}}{2{b}_{2}}{\varOmega\:}_{P}\left(0\right)\frac{{A}_{P}}{{A}_{s}}$$

Giving,

$$\:A=\frac{{a}_{2}}{{b}_{2}}{\varOmega\:}_{P}\left(0\right)\left[1+\frac{{a}_{1}}{2{b}_{2}}\frac{{A}_{P}}{{A}_{s}}\right]$$

Then,

$$\:{{\Omega\:}}_{\text{s}}=\frac{{a}_{2}}{{b}_{2}}{{\Omega\:}}_{\text{P}}\left(0\right)\left[1+\frac{{a}_{1}}{2{b}_{2}}\frac{{A}_{P}}{{A}_{s}}\right]{e}^{i{A}_{s}{b}_{2}Z}-\frac{{a}_{2}{a}_{1}}{2{b}_{2}}{\varOmega\:}_{P}\left(0\right){e}^{-i{A}_{s}{b}_{2}Z}\frac{{A}_{P}}{{A}_{s}}-\frac{{a}_{2}}{{b}_{2}}{{\Omega\:}}_{\text{P}}\left(z\right)$$

Results in,

A–15 $$\:{{\Omega\:}}_{\text{s}}\left(z\right)=\frac{{a}_{2}}{{b}_{2}}{{\Omega\:}}_{\text{P}}\left(0\right){e}^{i{A}_{s}{b}_{2}Z}+\frac{{a}_{2}{a}_{1}}{{b}_{2}^{2}}{\varOmega\:}_{P}\left(0\right)i\text{sin}\left({A}_{s}{b}_{2}Z\right)\frac{{A}_{P}}{{A}_{s}}-\frac{{a}_{2}}{{b}_{2}}{{\Omega\:}}_{\text{P}}\left(z\right)$$

Substitute Eq. (A–13) into (A–15) results in,

$$\:{{\Omega\:}}_{\text{s}}\left(z\right)\left[1-\frac{{b}_{1}{a}_{2}}{{a}_{1}{b}_{2}}\right]=\frac{{a}_{2}}{{b}_{2}}{{\Omega\:}}_{\text{P}}\left(0\right)\left[{e}^{i{A}_{s}{b}_{2}Z}-1\right]+\frac{{a}_{2}}{{b}_{2}}i{{\Omega\:}}_{\text{P}}\left(0\right)\left[\frac{{a}_{1}}{{b}_{2}}\frac{{A}_{P}}{{A}_{s}}\text{sin}\left({A}_{s}{b}_{2}Z\right)-\frac{{b}_{1}{a}_{2}}{{a}_{1}^{2}}\frac{{A}_{s}}{{A}_{P}}i\:\text{s}\text{i}\text{n}\left({A}_{P}{a}_{1}Z\right)\right]$$

Which finally results in

A–16 $$\begin{aligned} & \:\Omega \:_{s} \left( z \right) = \frac{{a_{1} b_{2} }}{{\left[ {a_{1} b_{2} - b_{1} a_{2} } \right]}}\Omega \:_{P} \left( 0 \right)\left[ {e^{{iA_{s} b_{2} Z}} - 1} \right] + \\ & \frac{{a_{1} b_{2} }}{{\left[ {a_{1} b_{2} - a_{2} b_{1} } \right]}}i\Omega \:_{P} \left( 0 \right)\left[ {\frac{{a_{1} }}{{b_{2} }}\sin \left( {A_{s} b_{2} Z} \right)\frac{{A_{P} }}{{A_{s} }} - \frac{{b_{1} a_{2} }}{{a_{1}^{2} }}\frac{{A_{s} }}{{A_{P} }}i\:\sin \left( {A_{P} a_{1} Z} \right)} \right] \\ \end{aligned}$$

## Appendix B: momentum matrix elements

Only two density matrix equations relating to the transitions considered in the SGC appear in this work. Although of this, all the transition momenta are calculated through this work. They are also used in the calculations. Their use is in the calculation of the transition coefficients $\:{\beta\:}_{ij}$ ($\:=\frac{{A}_{ij}}{2}+\frac{1}{{\tau\:}_{t}})$, with $\:{A}_{ij}(=\frac{{{\mu\:}_{ij}}^{2}{\omega\:}_{ij}^{2}}{3\pi\:\hslash\:{\epsilon\:}_{s}{c}^{3}})$ where $\:{\mu\:}_{ij}$ is the transition momentum between $\:\left| i \right\rangle$ and $\:\left| j \right\rangle$ states. Then, one has either QD-QD interdot transitions or WL-QD type depending on the states $\:\left| i \right\rangle$ and $\:\left| j \right\rangle$ are for DQD or WL. For the interdot transitions, taking $\:{\mu\:}_{02}$ momentum as an example^12^,

B–1 $$\:{\mu\:}_{02}={C}_{mn}\left\{{\int\:}_{0}^{a}{J}_{m}\left({p}_{0}\rho\:\right){J}_{m}\left({p}_{2}\rho\:\right)e{\rho\:}^{2}d\rho\:\}{\int\:}_{0}^{{h}_{d}}[\text{cos}\left({k}_{{z}_{0}}z\right)\text{cos}\left({k}_{{z}_{2}}z\right)\right]dz{\int\:}_{0}^{2\pi\:}\frac{1}{2\pi\:}d\varphi$$

In Eq. (B–1), $\:{C}_{mn}$ is the normalization constant for the in-plane QD wavefunction, which is in the form of the Bessel function $\:{J}_{m}\left({p}_{i}\rho\:\right)$ with $\:{p}_{i}$ its variable determined from the interface boundary conditions between the quantum disk and the WL material, $\:\rho\:$ is the radius of the quantum disk (QD). The wavenumber of the state $\:\left| i \right\rangle$ in the z-direction is$\:\:{k}_{{z}_{i}}$.

As an example of the WL-QD transitions, take the transition momentum $\:{\mu\:}_{35}$ in the VB, it is written as^12^,

B–2 $$\:\mu \:_{{35}} = \left\langle {\phi \:_{{QD}}^{{j = 3}} |e\vec{r}|\phi \:_{{WLv}} } \right\rangle$$

B–3 $$\:\mu \:_{{35}} = \left\langle {\psi \:_{{QD}}^{{j = 3}} |e\widehat{{\rho \:}}\rho \:|\psi \:_{{WLv}} } \right\rangle A_{{QD_{{z3}} }} A_{{w_{{z5}} }} \int \: \cos \left( {k_{{z_{v} }} z} \right)\cos \left( {k_{{zw_{v} }} z} \right)dz$$

There are two parts of integration here. The first is in the in-plane ($\:\rho\:$) direction of the quantum disk, and the second ( the cosine-type) is in the z-direction. This form is to cover the overall wavefunctions of the states under calculation. $\:\widehat{\rho\:}$ is a unit vector in the in-plane direction, the wavefunctions $\:{\phi\:}_{QD}^{j=3}$, $\:{\phi\:}_{WLv}$ are for QD state $\left| 3 \right\rangle$ and WL VB, in the $\:\rho\:$-direction, $\:{A}_{{QD}_{z3}}$, $\:{A}_{{w}_{z5}}$ are the normalization constants of the wavefunctions in the z-direction, while $\:{k}_{{z}_{v}}$ and $\:{k}_{{zw}_{v}}$ are the z-direction wavevectors for the QD state $\left| 3 \right\rangle$ and WL VB state, respectively. The first integration in Eq. (B–3), which is in the $\:\rho\:$-direction, contains the OPW integration. It is written as^15^,

B–4 $$\left| {\phi \:_{{QD}}^{{j = 3}} |e\widehat{{\rho \:}}\rho \:|\psi \:_{{WLv}} } \right\rangle \: = \frac{1}{{N_{{WL}} }}[\left| {\phi \:_{{QD}}^{{j = 3}} |e\rho \:|\psi \:_{{WLv}} } \right\rangle - \sum {\:_{{i = 0}}^{3} } \left\langle {\phi \:_{{QD}}^{{j = 3}} |e\rho \:|\phi \:_{{QD}}^{i} } \right\rangle \left\langle {\phi \:_{{QD}}^{i} |\phi \:_{{WLv}} } \right\rangle$$

$\:{\text{N}}_{\text{W}\text{L},\text{j}}$ is the normalization constant in the OPW, it is defined by^15^,

B–5 $$\:N_{{WL}} = \sqrt {1 - \left| {\sum {\:_{i} } \left\langle {\phi \:_{{QD}}^{i} |\phi \:_{{WL}} } \right\rangle } \right|^{2} }$$

The summation runs through all DQD states. Other integrations in Eq. (B–4) are written as^15^,

B–6 $$\left| {\phi \:_{{QD}}^{{j = 3}} |e\rho \:|\psi \:_{{WLv}} } \right\rangle \: = \frac{{C_{{mn}} \left| e \right|}}{{\sqrt A }}\int \: J_{{m,j}} \left( {p\rho \:} \right)e^{{ik\rho \:}} \rho \:^{2} d\rho$$

B–7 $$\left| {\phi \:_{{QD}}^{{j = 3}} |e\rho \:|\phi \:_{{QD}}^{{i = 2}} } \right\rangle \: = C_{{mn,j}} C_{{mn,i}} \left| e \right|\int_{0}^{{h/2}} {J_{{m,j}} \rho \:J_{{m,i}} \rho \:d\rho }$$

B–8 $$\left| {\phi \:_{{QD}}^{i} |\phi \:_{{WLv}} } \right\rangle \: = \frac{{C_{{mn}} }}{{\sqrt A }}\int \: J_{{m,j}} \left( {p\rho \:} \right)e^{{ik\rho \:}} \rho \:^{2} d\rho$$

A similar integration is also found for the CB.

## Appendix C: including incoherent pumping

The density matrix equations of the DQD-MNP under considering an incoherent pumping $\:{(R}_{inc})$ reads,

$$\begin{aligned} \:\mathop {\rho \:}\limits^{.} \:_{{20}} = & - \left[ {i\Delta \:_{{20}} + \left( {\gamma \:_{0} + \gamma \:_{2} } \right) + R_{{inc}} } \right]\rho \:_{{20}} + \left( {i\Omega \:_{{21}} \rho \:_{{10}} } \right) + \left( {\frac{{i\:sgc\:R_{{inc}} }}{2}\rho \:_{{30}} } \right) - \left( {iT_{{10}} \rho \:_{{21}} } \right) \\ & - \left( {i\Omega \:_{{30}} \rho \:_{{23}} } \right) - \left( {i\beta \:_{{40}} \rho \:_{{24}} } \right) - \left( {i\beta \:_{{52}} \rho \:_{{25}} } \right) + i\Omega \:_{{20}} \left( {\rho \:_{{00}} - \rho \:_{{22}} } \right) \\ \end{aligned}$$

$$\:\dot{\rho\:}{\:}_{30}=-\left[\left({\gamma\:}_{0}+{\gamma\:}_{2}\right)+{R}_{inc}\right]+i{{\Omega\:}}_{30}\left({\rho\:}_{00}{-\rho\:}_{33}\right)+\frac{i\:sgc\:{R}_{inc}}{2}{\rho\:}_{20}-{i\varOmega\:}_{20}{\rho\:}_{32}-{i\varOmega\:}_{20}{\rho\:}_{34}$$

Then, $\:{R}_{inc}$ is introduced into the following variables,

$$\:{A}_{d30}=\left({\gamma\:}_{0}+{\gamma\:}_{3}+{R}_{inc}\right)\left({\gamma\:}_{4}+{\gamma\:}_{3}\right)$$

$$\:{A}_{z20}=\frac{\left({\gamma\:}_{1}+{\gamma\:}_{2}\right)\left(i{\varDelta\:}_{20}+{\gamma\:}_{0}+{\gamma\:}_{2}+{R}_{inc}\right)}{\left({\gamma\:}_{1}+{\gamma\:}_{2}\right)\left(i{\varDelta\:}_{20}+{\gamma\:}_{0}+{\gamma\:}_{2}+{R}_{inc}\right)+{T}_{10}^{2}}$$

$$\:{A}_{d20}=\frac{1}{(i{\varDelta\:}_{20}+{\gamma\:}_{0}+{\gamma\:}_{2}+{R}_{inc})}$$

$$\:{b}_{1}=\frac{-{A}_{z20}{A}_{d20}\left(\frac{sgc\:{R}_{inc}}{2}\right)\:}{\left({\gamma\:}_{^\circ\:}+{\gamma\:}_{3}+{R}_{inc}\right)*{Add}_{20}}$$

$$\:{Add}_{20}=[1-{A}_{z20}{A}_{d20}\left(\frac{i\:sgc\:{R}_{inc}}{2}\right)\frac{1}{{A}_{d30}}\left\{\left(\frac{i\:sgc\:{R}_{inc}}{2}\right)\left({\gamma\:}_{4}+{\gamma\:}_{3}\right)-\left({\beta\:}_{40}{T}_{10}\right)\right\}$$
